# Pharmacophore Modelling and Synthesis of Quinoline-3-Carbohydrazide as Antioxidants

**DOI:** 10.1155/2011/592879

**Published:** 2011-02-14

**Authors:** Mustapha El Bakkali, Lhassane Ismaili, Isabelle Tomassoli, Laurence Nicod, Marc Pudlo, Bernard Refouvelet

**Affiliations:** ^1^Laboratoire de Biologie Cellulaire Equipe 2SBP EA4267, 4 Place Saint Jacque 25030 Besançon Cedex, France; ^2^Laboratoire de Chimie Thérapeutique Equipe 2SBP EA4267, 4 Place Saint Jacque 25030 Besançon Cedex, France

## Abstract

From well-known antioxidants agents, we developed a first pharmacophore model containing four common chemical features: one aromatic ring and three hydrogen bond acceptors. This model served as a template in virtual screening of Maybridge and NCI databases that resulted in selection of sixteen compounds. The selected compounds showed a good antioxidant activity measured by three chemical tests: DPPH radical, OH° radical, and superoxide radical scavenging. New synthetic compounds with a good correlation with the model were prepared, and some of them presented a good antioxidant activity.

## 1. Introduction

Free radicals play an important role in the pathogenesis of many diseases, accounting for continuing interest in the identification and development of novel antioxidants that prevent radical-induced damage.

In humans, several pathologies involve the overproduction of reactive oxygen species (ROS): these oxygen species such as the superoxide radical anion (O_2_^−°^) and hydrogen peroxide (H_2_O_2_) are formed by the partial reduction of molecular oxygen. Formation of the hydroxyl radical (HO°), another ROS, is thought to occur through the one-electron reduction of H_2_O_2_. This reaction is facilitated by transition metals that are in a reduced valence state (e.g., reduced copper or iron) [1]. Additionally, there are a large number of other reactive species that are formed from the reaction ROS with biological molecules (e.g., polyunsaturated lipids, thiols, and nitric oxide (NO)) [2]. For example, O_2_^−°^ reacts with NO to form peroxynitrite anion (ONOO^−^), which is unstable at physiological pH and rapidly decomposes. It forms potent nitrating and oxidizing species [3, 4] or hypochlorite (XOCl) that is a powerful oxidant produced by activated neutrophils via the reaction of H_2_O_2_ and Cl^−^, catalysed by the heme enzyme myeloperoxidase [5].

A lot of natural and synthetic products like quercetin **1**, curcumine **2**, resveratrol **3**, Trolox **4,** and *N*-acetylcystein **5** are known for their antioxidant activity [6–10]. Some heterocyclic compounds, either natural (phytoestrogens) or obtained by synthesis, having coumarin or quinoline rings, were studied for their biological activity. They are used especially as radicals scavenger like quercetol and coumestrol [11, 12] or the copper or iron chelating molecules such as clioquinol [13, 14].

After first studies realized in our laboratory [15–17] on new compounds with quinoline and coumarin structures and with the aim of discovering a very strong antioxidant, we decided to introduce in our research the three-dimentional generation and database searching. The increasing number of successful applications of 3D-pharmacophore-based searching in medicinal chemistry clearly demonstrates its utility in the modern drug discovery paradigm [18, 19]. In the absence of such three-dimensional structure-based, we attempted to identify the hypothetical 3D-ligand-based pharmacophore model by using the common features hypothesis generation approach (HipHop) implemented in the program Catalyst [20]. In particular, HipHop algorithm finds common feature pharmacophore models among a set of highly active compounds and carry out a qualitative model (without taking care of the activity data) which represents the essential 3D arrangement of functional groups common to the set of molecules that explains the specific activity, antioxidant in the current study. 

 The generation of a pharmacophore model for antioxidant from a training set of five molecules using catalyst/HipHop gave ten hypothesis, the best one was used for the databases search. The identified compounds were tested and discussed to validate a pharmacophore hypothesis.

Then, this pharmacophore was used to predict and select the synthesis of new quinoline derivatives, many compounds were prepared and their antioxidant properties evaluated by hydroxyl radical °OH scavenging activity and by their antiradical activity against 2,2-diphenyl-1-picrylhydrazyl radical (DPPH^•^) and anion superoxide.

## 2. Result and Discussion

### 2.1. Training Set

**Scheme 1:**
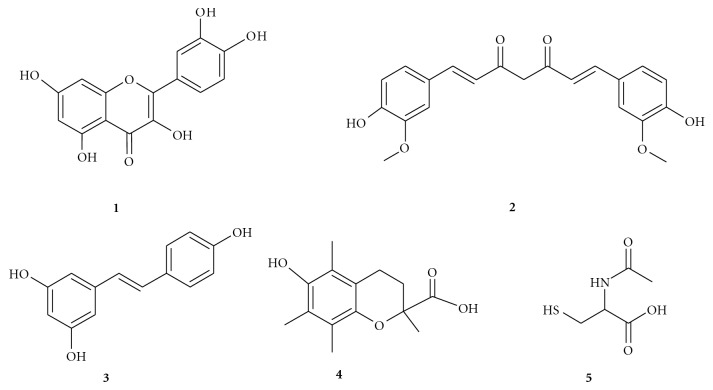
Chemical structure of Quercetin **1,** curcumine **2,** resveratrol **3, **Trolox **4**, and *N*-acetyl cystein **5.**

Five molecules Quercetin **1**, curcumine **2**, resveratrol **3**, Trolox **4**, and *N*-acetyl cystein **5** as shown in the Scheme 1 were selected for the training set representing the best known natural antioxidants [5–10]. All structures were generated using editor sketcher in DS Catalyst software package and to build conformational models of up to 250 conformers for each molecule, the “best conformer generation” option and 10 kcal/mol energy cutoff were chosen.

### 2.2. Pharmacophore Model Generation

Our Pharmacophoric analysis was carried out using the Catalyst/HipHop procedure to evaluate the common feature required and the hypothetical geometries of these ligands in their most active forms. 

In the hypothesis generation based on the atom types in the molecules of the training set, the following chemical functions were selected in the feature dictionary of Catalyst: Hydrogen bond acceptor, hydrogen bond donor, aromatic ring, positive ionisable and hydrophobic groups.

Ten hypothesis (Hypo 1 to Hypo 10) were obtained using the default parameters of catalyst. These hypothesis had scores from 33.52 to 36.68 (Table 1) so we studied if they mapped to all the important features of the active compound, we searched the correlation between best values, conformational energies, and activity of the training set (data not shown) and we selected the highest ranked pharmacophore hypothesis (Hypo1) for the database search.

**Figure 1:**
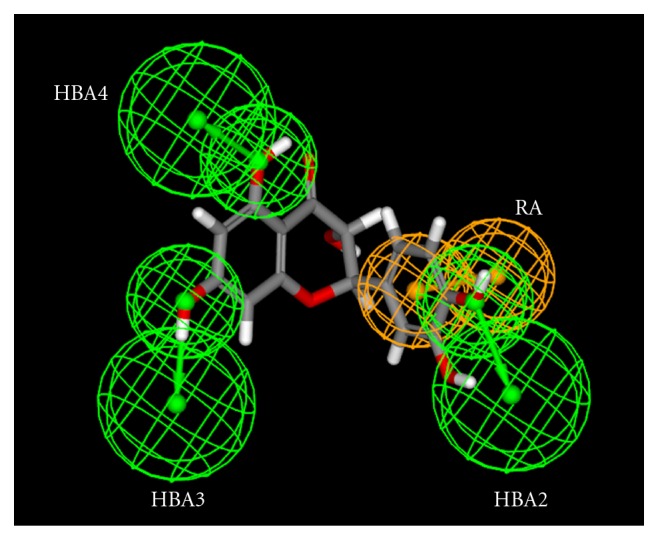
Quercetin mapping with selected pharmacophore.

This selected pharmacophore model contains four chemical features: one aromatic ring (RA) (orange colour) and three hydrogen bond acceptors (HBA2, HBA3 and HBA4) (green colour). The RA maps the aromatic ring attached to position 2 of benzopyrane group of quercetin, the HBA2 maps the hydroxyl group at position 4 of aromatic ring, HBA3 and HBA4 maps respectively the hydroxyl groups at position 7 and 5 as shown in Figure 1.This alignment represents a good match of features of the pharmacophore model with the ligand (fit value = 3.99/4). 

**Scheme 2:**
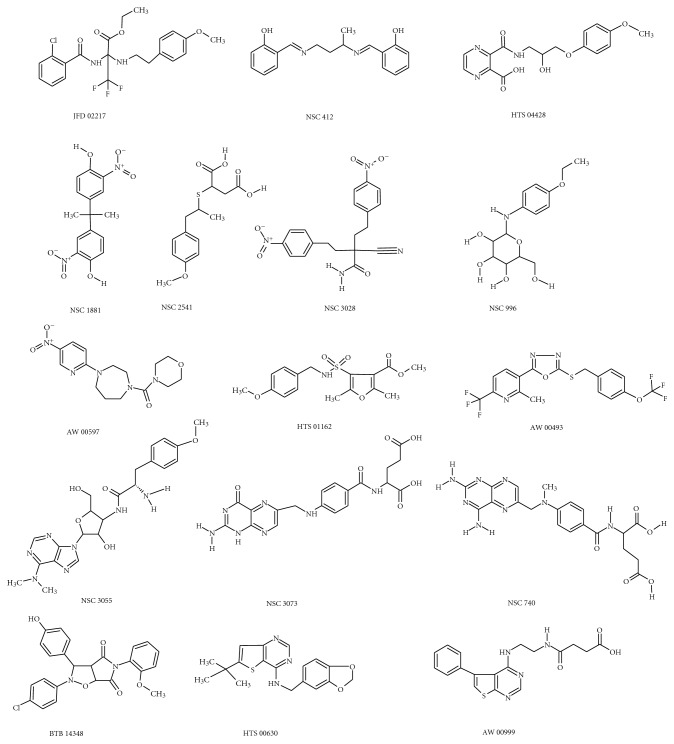
Chemical structure of selected sixteen compounds.

We employed this model as 3D-search query against the NCI, Maybridge, and minimaybridge structure databases (each contained thousands of compounds) using the “fast flexible search” approach implemented within Catalyst. The pharmacophore captured 300 hits for each database, we selected sixteen compounds Scheme 2 on the basis of fit value Log *P* and availability.

### 2.3. Antioxidant Activities of Identified Compounds

Free radical scavenging is one of the best known mechanisms by which antioxidants inhibit lipid oxidation. DPPH, Superoxide, and hydroxyl radical scavenging activity evaluation are standard assays in antioxidant activity studies and offer rapid techniques for screening the radical scavenging activity (RSA) of specific compounds. The stable free radical 2;2-diphenyl-1-picrylhydrazyl (DPPH) is a useful reagent to investigate the scavenger properties of polyphenols. It is now widely accepted that the reaction between phenols and DPPH proceeds through two different mechanisms: The direct hydrogen atom transfer (HAT) and the sequential proton less electron transfer. Superoxide anion produced by activated human neutrophils can be a source of additional harmful ROS and no radical species as the singulet oygen in vivo. The hydroxyl radical is considered the most damaging free radical for living cells because it leads to deleterious oxidations of cellular components including protein, DNA and lipids. The RSA of 16 identified compounds was estimated using these three methods. 

#### 2.3.1. DPPH Radical Scavenging

**Scheme 3:**
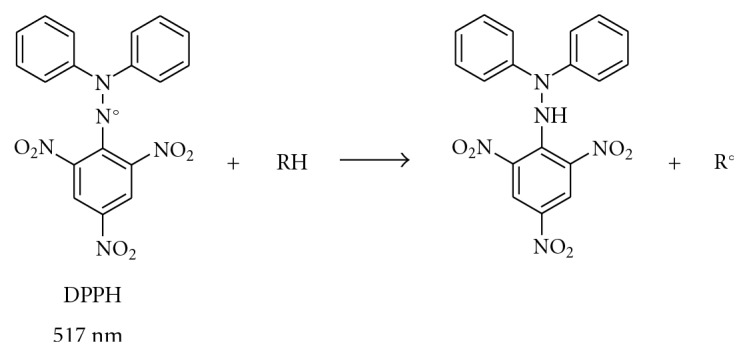
Reactivity of DPPH radical.

A freshly prepared DPPH solution exhibits a deep purple colour with a maximum absorption at 517 nm. This purple colour generally disappears when an antioxidant is present in the medium as shown in Scheme 3. Thus, antioxidant molecules can quench DPPH free radicals (by providing hydrogen atoms or by electron donation, conceivably via a free-radical attack on the DPPH molecule) and convert them to colourless/bleached product [21, 22].

The RSA against DPPH radical of 16 identified molecules were examined and compared (Table 2). Results are expressed as a percentage of the ratio of the decrease in absorbance at 517 nm, to the absorbance of DPPH solutions in the absence of compounds at 517 nm.

#### 2.3.2. OH° Radical Scavenging

We used the benzoic acid method [23]. The benzoic acid was hydroxylated by OH° formed by Fenton reaction at C3 or C4 positions of the aromatic ring and the fluorescence was measured at 407 nm emission with excitation at 305 nm. This fluorescence generally decreases when an antioxidant is present in the medium. Antioxidant molecules prevent the hydroxylation of benzoic acid by providing hydrogen atom.

The RSA OH° result of molecules identified were summarized in (Table 2), this results are expressedas

(1) $$\;\begin{array}{cc} \text{RSA}\;{\text{OH}}^{{}^{\circ}}\text{\%} & =\frac{\left[\text{Absorbance}\;\text{in}\;\text{the}\;\text{presence}\;\text{of}\;\text{sample}\right]}{\left[\text{Absorbance}\;\text{in}\;\text{the}\;\text{absence}\;\text{of}\;\text{sample}\right]}\\ & \;\times 100. \end{array}$$

#### 2.3.3. Superoxide Radical Scavenging

Superoxide radical scavenging activity was determined by absorbance measurement of the blackish blue formazan product by superoxide addition to nitro blue tetrazolium (NBT) substrate, according to the method of Nishikimi et al. [24]. Superoxide was generated chemically by the reduction of phenazine methosulfate (PMS), using *β*-NADH as the electron donor in the presence of dissolved molecular oxygen in the reaction solution. 

The RSA O^2−^ results in molecules identified were summarized in Table 2. The percentage scavenging effects were calculated from the decrease in absorbance against control. This absorbance was measured at 560 nm.

From analysis of Table 2, we can conclude that all the identified compounds present a scavenging effect. For The results RSA of DPPH radicals, seven compounds **(AW** **00493**, **BTB** **14348**, **HTS** **0630**, **NSC** **2541**, **NSC** **3028**, **NSC** **412**, **and** **NSC** **740)** have the same or better activity than the standard N-acetylcysteine at 50 *μ*mol·L^−1^. 

Based on the result of superoxide radical scavenging we demonstrated that all compounds show a dose-dependent effect. 

Concerning the RSA of radical hydroxyl, all compounds have same or better results than standards at 50, 100, and 150 *μ*mol·L^ −1^

These results show that the theoretical pharmacophore has got a discriminant power. It allows the selection of antioxidants molecules from a databasis containing thousand of compounds.

## 3. Synthesis

We envisaged doing the synthesis of compounds presenting a good correlation with the pharmacophore established to verify if it allows predicting the activity of molecules. We chose the quinoline derivatives to continue the work already realized on the synthesis of new quinoline derivatives by our laboratory [15–17]. 

Different molecules were proposed and first mapped on selected pharmacophore using ligand pharmacophore protocols. For these compounds, we obtained fit values from 2.6 to 3.3

**Figure 2:**
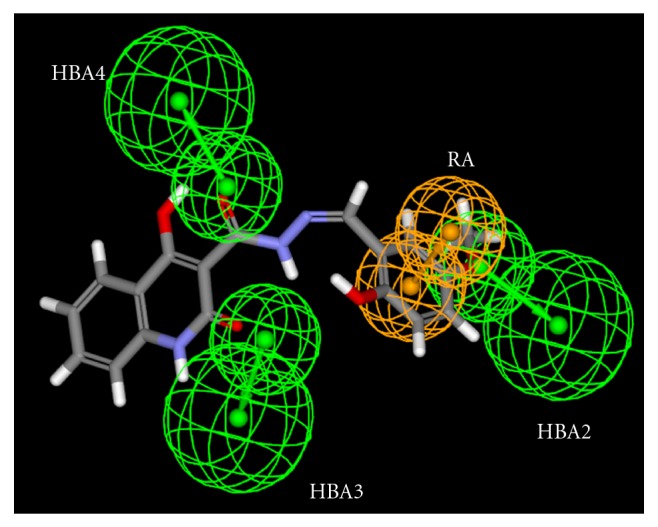
Compound 8c mapping with a pharmacophore.

In Figure 2 we present the compound 8c (fit value = 3.3/4) mapping with a previously selected pharmacophore, we can see that the RA maps the aromatic ring of phenolic group, the HBA2 maps the hydroxyl group at position 5 of phenolic group, HBA3 and HBA4 map respectively the carbonyl groups at position 2 of quinoline and carbonyl of carbohydrazide. 

**Scheme 4:**
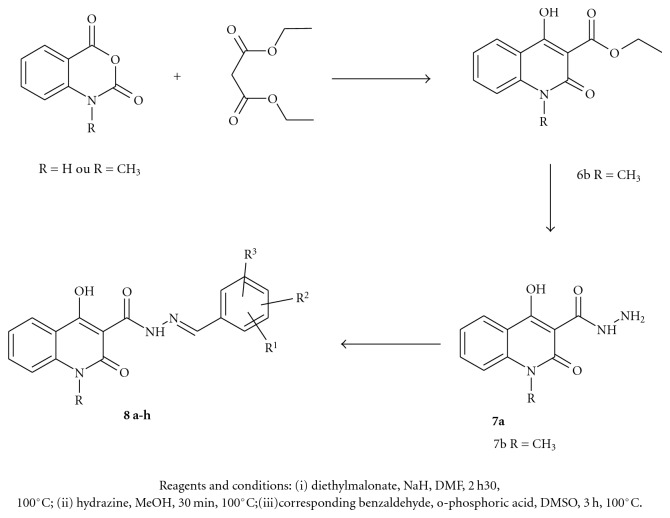
Synthetic route to prepare compounds **8.**

The synthetic route to prepare desired substituted 4-hydroxy-2-oxophenylmethylene-1,2-dihydroquinolin-3-carbohydrazide is described in Scheme 4. 

The condensation N-H or N-methyl anhydride isatoique with ethylmalonate in dimethylformamide gave ethyl 4-hydroxy-2-oxo-1,2-dihydroquinoline-3-carboxylate **6a** or his derivatives N-methyl **6b **[25]. The **6a **and 6d were converted in resulting **7a** and **7b** with hydrazine hydrate in methanol, finally they reacted with different aldehydes. This procedure gave compounds **8a–c**, as a mixtures of *E*-and *Z*-isomers in a *E* *:* *Z *: 9 : 1, 7 : 3 and 4 : 1 ratio, respectively, whereas the target molecules **8d–h** were isolated as pure *E* isomers. The *E* configuration compounds **8 **was characterized in the 2D-NMR (^1^H-^1^H) spectra by NOESY experiments, and analyzing by the NOE effects on the hydrogens for the N*H* amide of carbohydrazide moiety, and the C*H* of imines. 

All the compounds summarized in (Table 3) were obtained in moderate to good yields ranging from 56% to 94%. All these products were isolated from reaction mixture by recrystallisation from ethanol, and their structures were characterized by ^1^H NMR, IR spectra and elementary analysis.

The antioxidant activity for these compounds was measured by two methods DPPH and anion superoxide (Table 4), the hydroxyl radical scavenging is not applicable for these compounds because of their fluorescence at the studied wavelength (407 nm emission with excitation at 305 nm).

All synthesized compounds exhibit antiradical activity against DPPH radical and anion superoxide tests. The products 8a and 8c having a good fit value present better results. The product 8h has a lower result. It's probably due to absence of hydroxyl group

**Conclusion 3 .**

The present study is a successful example for a rational identification of antioxidants agents. This was accomplished by generating a three-dimensional pharmacophore model based on a training set of five well-know antioxidants. The model containing one aromatic group and three hydrogen bond acceptors was selected and used to identify new quinoline derivatives antioxidant agents.

## 4. Experimental

### 4.1. Antioxidant Activity Studies

**Assay of Hydroxyl Radical (OH°) Scavenging Activity**

In a screw-capped test tube, 0.2 mL of sodium benzoate (10 mmol), 0.2 mL of FeSO_4_·7H_2_0 (10 mmol) and EDTA (10 mmol) were added. Then the sample solution and a phosphate buffer (pH 7.4, 0.1 mol) were mixed to give a total volume of 1.8. Finally, 0.2 mL of H_2_O_2_ solution (10 mmol) was added, and the whole incubated at 37°C for 2 h. After incubation, the fluorescence was measured on spectrofluorimeter Shimadzu RF 10AXL at wavelengths 407 nm for emission and 305 nm for excitation.

**DPPH Radical Scavenging Activity**

The capacity of compounds to scavenge the “stable” free radical DPPH was monitored according to the method of Hatano et al. [26]. Various concentrations of methanolic compounds solutions (0.3 mL) were mixed with methanolic solution containing DPPH radicals (1.5·10^−4^ M, 2.7 mL). The mixture was shaken vigorously and left to stand for 2 h in the dark (until stable absorption values were obtained). The reduction of the DPPH radical was determined by measuring the absorbance at 517 nm. The RSA was calculated as a percentage of DPPH colouration using

(2) $$\text{\%}\;\text{RSA}\;=\left[\frac{\left(A_{\text{DPPH}}-A_{S}\right)}{A_{\text{DPPH}}}\right]\times 100,$$

where *A*_*S*_ is the absorbance of the solution when the compound has been added at a particular level and *A*_DPPH_ is the absorbance of the DPPH solution. Mean values from three independent samples were calculated for each compound and standard deviations were less than 5%.

**O_2_^−^ Radical Scavenging Activity**

The reaction mixture (1 mL) contained 700 *μ*L of various concentrations of methanolic compounds solutions, 100 *μ*L of *β*-NADH (1 mM in water), 100 *μ*L of NBT (1 mM in 1 M-phosphate buffer, pH 7.8 and 100 *μ*L of PMS (120 *μ*M in water) added in that order and the mixture allowed to react at RT for 10 min. The control contained all the reaction reagents except the test material. The reaction was terminated by adding 40 *μ*L of concentrated HCl (10 mM) and absorbance was measured at 560 nm against blanks that contained all compound except test material and PMS.

The percentage scavenging effects was calculated from the decrease in absorbance against control.

### 4.2. Computational Methods

All molecular modelling studies were performed using discovery studio 2.1 with catalyst module. All structures were generated using 2D/3D editor sketcher and minimized to the closest minimum using the CHARMm-like force field implemented in the program [27]. A stochastic research coupled to a poling method [28] was applied to generate conformers for each compound by using “Best conformer generation” option with a 20 kcal/mol energy cutoff (20 kcal/mol maximum compared to the most stable conformer). 

 The pharmacophore-based investigation involved using the catalyst/Hip/Hop program to generate feature based 3D pharmacophore alignments [29]. This was performed in a three step procedures: (a) a conformation model of each molecule in the training set was generated, (b) each conformer was examined for the presence of certain chemical features, (c) a three dimensional configuration of chemical feature these steps were performed with a module common feature pharmacophore generation.

### 4.3. Synthesis

#### 4.3.1. General Methods

Reactions were monitored by TLC using precoated silica gel aluminum plates containing a fluorescent indicator (Macherey-Nagel). Detection was done with UV (254 nm). Melting points were determined on a Kofler block and were uncorrected. Infrared spectra were recorded on a Shimadzu FTIR-8201 PC spectrometer in KBr (*ν* in cm^−1^). ^1^H NMR spectra were recorded on a Bruker AC 300 spectrometer. Microanalyses were carried out by the Service Central d'Analyses, CNRS, Vernaison (France). All reagents were pure analytical grades and used without further purification.

#### 4.3.2. General Method of Preparation of Compounds **6a-b**

The corresponding anhydride isatoic (1 eq) was suspended in DMF (10 mL) at 0°C. Sodium hydride (2 eq) and diethyl malonate (5 eq) were added slowly. The reaction mixture was heated at 85°C for 5 h. Then, 10 mL of water were added and the mixture was acidified with concentrated hydrochloric acid. The resulting solid was filtered, washed with water and dried, yielding the desired compound.

#### 4.3.3. Ethyl 4-hydroxy-2-oxo-1,2-dihydroquinoline-3-carboxylate **(6a)**

Following the General procedure in Section 4.3.2, the reaction of isatoic anhydride (1 g, 6.13 mmol) with NaH (0.29 g, 12.3 mmol), and diethyl malonate (4.91 g, 30.7 mmol) gave product **6a **(1 g, 70%): mp 134°C; IR (KBr) *υ* 3406, 3193, 1658, 1604 cm^−1^; ^1^H NMR (DMSO-*d*_6_, 300 MHz) *δ* 11.47 (s, 1H), 7.94 (d, *J* = 8.1 Hz, 1H), 7.62 (t, *J* = 7.2 Hz, 1H), 7.27 (d, *J* = 8.1 Hz, 1H), 7.20 (t, *J* = 7.5 Hz, 1H), 4.34 (q, *J* = 6.9 Hz, 2H), 1.31 (t, *J* = 7.2 Hz, 3H). Anal. Calcd. for C_12_H_11_NO_4_: C, 61.80%; H, 4.75%; N, 6.01%. Found: C, 61.72%; H, 4.78%; N, 6.10%.

#### 4.3.4. Ethyl 4-hydroxy-1-methyl-2-oxo-1,2-dihydroquinoline-3-carboxylate **(6b)**

Following the General procedure in Section 4.3.2, the reaction of *N*-methyl anhydride isatoic (1 g, 5.65 mmol) with NaH (0.27 g, 11.3 mmol), and diethyl malonate (4.52 g, 28.2 mmol) gave product **6b** (0.56 g, 41%): mp 104°C; IR (KBr) *υ* 1631, 1593, 1562 cm^−1^; ^1^H NMR (DMSO-*d*_6_, 300 MHz) *δ* 8.05 (d, *J* = 7.8 Hz, 1H), 7.45 (t, *J* = 7.5 Hz, 1H), 7.52 (d, *J* = 8.4 Hz, 1H), 7.31 (t, *J* = 7.2 Hz, 1H), 4.33 (q, *J* = 7.2 Hz, 2H), 3.54 (s, 3H), 1.30 (t, *J* = 7.2 Hz, 3H). Anal. Calcd. for C_13_H_13_NO_4_: C, 63.15%; H, 5.30%; N, 5.67%. Found: C, 63.24%; H, 5.27%; N, 5.61%.

#### 4.3.5. General Method for Compounds **7a-b**

The quinoline-3-carboxylate (1 eq) and its derivatives were suspended in methanol (20 mL). hydrazine (1.5 eq) was added and the mixture was heated at 100°C for 30 min. The precipitated compound was collected by filtration and used without further purification.

#### 4.3.6. Hydroxy-2-oxo-1,2-dihydroquinoline-3-carbohydrazide **(7a)**

Following the General procedure in Section 4.3.5 the reaction of hydrazine (0.52 g, 16.3 mmol) with compound **6a** (2 g, 8.58 mmol) gave product **7a** (1.57 g, 84%): mp > 260°C; IR (KBr) *υ* 3167, 1674, 1616, 1531 cm^−1^; ^1^H NMR (DMSO-*d*_6_, 300 MHz) *δ* 11.89 (s, 1H), 10.97 (s, 1H), 7.97 (d, *J* = 7.9 Hz, 1H), 7.68 (t, *J* = 7.2 Hz, 1H), 7.36 (d, *J* = 8.3 Hz, 1H), 7.29 (t, *J* = 7.3 Hz, 1H), 2.79 (s, 2H). Anal. Calcd. for C_10_H_9_N_3_O_3_: C, 54.79%; H, 4.14%; N, 19.17%. Found: C, 54.85%; H, 4.08%; N, 19.90%.

#### 4.3.7. 4-Hydroxy-1-methyl-2-oxo-1,2-dihydroquinoline-3-carbohydrazide **(7b)**

Following the General procedure in Section 4.3.5 the reaction of hydrazine (0.48 g, 15 mmol) with compound **6b** (2 g, 8.10 mmol) gave product **7b** (1.20 g, 64%): mp > 260°C; IR (KBr) *υ* 3328, 3240, 1647, 1589 cm^−1^; ^1^H NMR (DMSO-*d*_6_, 300 MHz) *δ* 11.00 (s, 1H), 8.01 (d, *J* = 7.2 Hz 1H), 7.81 (t, *J* = 7.3 Hz, 1H), 7.62 (d, *J* = 8.6 Hz, 1H), 7.38 (t, *J* = 7.3 Hz, 1H), 4.95 (s, 2H), 3.63 (s, 3H). Anal. Calcd. for C_11_H_11_N_3_O_3_: C, 56.65%; H,4.75%; N, 18.02%. Found: C, 56.52%; H, 4.79%; N, 18.11%.

#### 4.3.8. General Method for Compounds **(8a–h)**

The corresponding quinoline-3-carboxyhydrazides **7a**-**b** were stirred with 2,4-dihydroxybenzaldehyde or its derivatives in dimethyl sulfoxide and four drops of orthophosphoric acid for 15 min at room temperature. The mixture was then heated at 100°C for 1 h. The compound was collected by filtration and washed with water.

#### 4.3.9. *N′*-[(*E*)-(2,4-Dihydroxyphenyl)methylidene]-4-hydroxy-2-oxo-1,2-dihydroquinoline-3-carbohydrazide **(8a)**

Following the General procedure in Section 4.3.8, the reaction of 2,4-dihydroxybenzaldehyde (0.31 g, 2.3 mmol) with compound **7a** (0.5 g, 2.3 mmol) gave product **8a** (0.75 g, 97%): mp > 260°C; IR (KBr) *υ* 3205, 1658, 1558 cm^−1^; ^1^H NMR (DMSO-*d*_6_, 300 MHz) *δ* 13.19 (s, 1H), 12.01 (s, 1H), 11.08 (s, 1H), 10.10 (s, 1H), 8.51 (s, 1H), 7.96 (d, *J* = 7.9 Hz, 1H), 7.66 (t, *J* = 7.7 Hz, 1H), 7.34 (t, *J* = 7.3 Hz, 2H), 7.27 (t, *J* = 7.7 Hz, 1H), 6.34 (d, *J* = 8.4 Hz, 1H), 6.29 (s, 1H). Anal. Calcd. for C_17_H_13_N_3_O_5_: C, 60.18%; H, 3.86%; N, 12.38%. Found: C, 60.24%; H, 3.84%; N, 12.31%.

#### 4.3.10. 4-Hydroxy-*N′*-[(1*E*)-(2-hydroxy-5-methylphenyl)methylene]-2-oxo-1,2-dihydroquinoline-3-carbohydrazide **(8b)**

Following the General procedure in Section 4.3.8, the reaction of 2-hydroxy-5-methyl-benzaldehyde (0.15 g, 1.10 mmol) with compound **7a** (0.25 g, 1.14 mmol) gave product **8b** (0.31 g, 81%): mp > 260°C, IR (KBr) *υ* 3001, 1651, 1612, 1581, 1546 cm^−1^; ^1^H NMR (DMSO-*d*_6_, 300 MHz) *δ* 13.65 (s, 1H), 8.87 (s, 1H), 8.27 (d, *J* = 7.9 Hz, 1H), 7.97 (t, *J* = 7.5 Hz, 1H) 7.67 (s, 1H), 7.57 (t, *J* = 7.5 Hz, 1H), 7.39 (d, *J* = 8.1 Hz, 1H), 7.11 (t, *J* = 8.6 Hz, 1H), 2.75 (s, 3H). Anal. Calcd. for C_18_H_15_N_3_O_4_: C, 64.09%; H, 4.48%; N, 12.46%. Found: C, 64.12%; H, 4.46%; N, 12.38%.

#### 4.3.11. 4-Hydroxy-*N′*-[(1*E*)-(2-hydroxy-5-methoxyphenyl)methylene]-2-oxo-1,2-dihydroquinoline-3-carbohydrazide **(8c)**

Following the General procedure in Section 4.3.8, the reaction of 2-hydroxy-5-methoxy-benzaldehyde (0.28 g, 1.84 mmol) with compound **7a** (0.40 g, 1.83 mmol) gave product **8c** (0.59 g, 92%): mp > 260°C; IR (KBr) *υ* 3355, 1670, 1651, 1577, 1542 cm^−1^; ^1^H NMR (DMSO-*d*_6_, 300 MHz) *δ* 13.41 (s, 1H), 10.43 (s, 1H), 8.92 (s, 1H), 8.58 (s, 1H), 7.98 (d, *J* = 7.9 Hz, 1H), 7.68 (t, *J* = 7.9 Hz, 1H), 7.36 (d, *J* = 8.3 Hz, 1H), 7.23 (m, 1H), 7.12 (d, *J* = 2.8 Hz, 1H), 6.90 (m, 3H), 3.69 (s, 3H). Anal. Calcd. for C_18_H_15_N_3_O_5_: C, 61.19%; H, 4.28%; N, 11.89%. Found: C, 61.26%; H, 4.25%; N, 11.82%.

#### 4.3.12. *N′*-[(1*E*)-(2,6-Dichloro-3-nitrophenyl)methylene]-4- hydroxy-2-oxo-1,2-dihydroquinoline-3-carbohydrazide **(8d)**

Following the General procedure in Section 4.3.8, the reaction of 2,6-dichloro-3-nitro-benzaldehyde (1 g, 4.55 mmol) with compound **7a** (0.50 g, 2.30 mmol) gave product **8d** (0.87 g, 92%): mp > 260°C; IR (KBr) *υ* 3298, 3093, 1735, 1620, 1596, 1539 cm^−1^; ^1^H NMR (DMSO-*d*_6_, 300 MHz) *δ* 13.92 (s, 1H), 11.80 (s, 1H), 8.60 (s, 1H), 8.16 (d, *J* = 8.5 Hz, 1H), 8.01 (d, *J* = 8.1 Hz, 1H), 7.88 (d, *J* = 8.7 Hz, 1H), 7.66 (s, 1H), 7.35 (s, 1H), 7.26 (s, 1H). Anal. Calcd. for C_17_H_10_Cl_2_N_4_O_5_: C, 48.48%; H, 2.39%; Cl, 16.83%; N, 13.30%. Found: C, 48.44%; H, 2.37%; Cl, 16.86%; N, 13.25%.

#### 4.3.13. *N′*-[(1*E*)-(2,6-Dichloro-3-nitrophenyl)methylene]-4-hydroxy-1-methyl-2-oxo-1,2-dihydroquinoline-3-carbohydrazide **(8e)**

Following the General procedure in Section 4.3.8, the reaction of 2,6-dichloro-3-nitro-benzaldehyde (0.78 g, 3.55 mmol) with compound** 7b** (0.30 g, 1.28 mmol) gave product **8e **(0.51 g, 91%): mp 230°C; IR (KBr) *υ* 3058, 1635, 1577, 1558, 1519 cm^−1^; ^1^H NMR (DMSO-*d*_6_, 300 MHz) *δ* 8.66 (s, 1H), 8.15 (d, *J* = 7.2 Hz, 2H), 7.87 (d, *J* = 8.7 Hz, 1H), 7.79 (s, 1H), 7.60 (s, 1H), 7.36 (s, 1H), 3.64 (s, 3H). Anal. Calcd. for C_18_H_12_Cl_2_N_4_O_5_: C, 49.67%; H, 2.78%; Cl, 16.29%; N, 12.87%. Found: C, 49.71%; H, 2.76%; Cl, 16.31%; N, 12.82%.

#### 4.3.14. 4-Hydroxy-*N′*-[(1*E*)-(2-hydroxy-5-methylphenyl)methylene]-1-methyl-2-oxo-1,2-dihydroquinoline-3-carbohydrazide **(8f)**

Following the General procedure in Section 4.3.8, the reaction of 2-hydroxy-5-methyl-benzaldehyde (0.15 g, 1.10 mmol) with compound **7b** (0.25 g, 1.07 mmol) gave product **8f** (0.34 g, 93%): mp > 260°C; IR (KBr) *υ* 2985, 1624, 1589, 1558, 1519 cm^−1^; ^1^H NMR (DMSO-*d*_6_, 300 MHz) *δ* 12.55 (s, 1H), 9.95 (s, 1H), 7.81 (s, 1H), 7.30 (d, *J* = 7.7 Hz, 1H), 7.00 (t, *J* = 8.1 Hz, 1H), 6.82 (d, *J* = 8.6 Hz, 1H), 6.58 (d, *J* = 7.5 Hz, 1H), 6.53 (s, 1H), 6.30 (d, *J* = 8.3 Hz, 1H), 6.01 (d, *J* = 8.3 Hz, 1H), 2.83 (s, 3H), 1.41 (s, 3H). Anal. Calcd. for C_19_H_17_N_3_O_4_: C, 64.95%; H, 4.88%; N, 11.96%. Found: C, 64.92%; H, 4.86%; N, 11.95%.

#### 4.3.15. 4-Hydroxy-*N′*-[(1*E*)-(2-hydroxy-5-methoxyphenyl)methylene]-1-methyl-2-oxo-1,2-dihydroquinoline-3-carbohydrazide **(8g)**

Following the General procedure in Section 4.3.8, the reaction of 2-hydroxy-5-methoxy-benzaldehyde (0.16 g, 1.05 mmol) with compound **7b** (0.25 g, 1.07 mmol) gave product **8g** (0.33 g, 84%): mp > 260°C; IR (KBr) *υ* 2966, 1643, 1562, 1535 cm^−1^: ^1^H NMR (DMSO-*d*_6_, 300 MHz) *δ* 13.49 (s, 1H), 10.51 (s, 1H), 8.65 (s, 1H), 8.15 (d, *J* = 7.5 Hz, 1H), 7.82 (d, *J* = 7.0 Hz, 1H), 7.65 (d, *J* = 8.1 Hz, 1H), 7.40 (t, *J* = 7.2 Hz, 1H), 7.15 (s, 1H), 6.92 (m, 2H) 3.74 (s, 3H), 3.67 (s, 3H). Anal. Calcd. for C_19_H_17_N_3_O_5_: C, 62.12%; H, 4.66%; N, 11.44%. Found: C, 62.19%; H, 4.63%; N, 11.41%.

#### 4.3.16. 4-Hydroxy-1-methyl-*N′*-[(1*E*)-(2-nitrophenyl)methylene]-2-oxo-1,2-dihydroquinoline-3-carbohydrazide **(8h)**

Following the General procedure in Section 4.3.8, the reaction of 2-nitrobenzaldehyde (0.78 g, 5.16 mmol) with compound **7b** (0.20 g, 0.86 mmol) gave product **8h** (0.29 g, 92%): mp > 260°C; IR (KBr) *υ* 1670, 1566, 1523 cm^−1^; ^1^H NMR (DMSO-*d*_6_, 300 MHz) *δ* 8.67 (s, 1H), 8.15 (s, 1H), 8.11 (d, *J* = 7.6 Hz, 1H), 8.07 (s, 1H), 7.83 (t, *J* = 6.9 Hz, 1H), 7.67 (m, 2H), 7.48 (s, 1H), 7.26 (s, 1H), 3.58 (s, 3H). Anal. Calcd. for C_18_H_14_N_4_O_5_: C, 59.02%; H, 3.85%; N, 15.29%. Found: C, 59.11%; H, 3.82%; N, 15.25%.

## Figures and Tables

**Table 1:** Summary of hypothesis.

Hypo	Feature	Rank	Direct hit mask	Partial hit mask
1	RAAA	36.68	01111	10000
2	RAAA	36.43	01111	10000
3	RAAA	36.29	01111	10000
4	AAAA	36.04	11101	00010
5	AAAA	35.82	11101	00010
6	RAAA	35.67	11111	10000
7	AAAA	35.19	11101	00010
8	HAAA	34.10	01111	10000
9	HAAA	33.70	01111	10000
10	HAAA	33.52	01111	10000

In direct hit mask, (1) indicates every feature of training set molecule is mapped, (0) indicates 1 or more features were not mapped.

In partial hit mask, (0) indicates every feature of training set molecule is mapped; (1) indicates 1 or more features were not mapped.

R: ring aromatic (RA), A: hydrogen bond acceptor (HBA), H: hydrophobic (H).

**Table 2:** DPPH radical, hydroxyl radical, and superoxide radical scavenging of identified compounds.

Compounds	Concentration *μ*mol·L^ −1^								
	50 *μ*mol·L^ −1^			100 *μ*mol·L^ −1^			150 *μ*mol·L^ −1^		

**%RSA∗**	DPPH	OH°	O_2_^−^	DPPH	OH°	O_2_^−^	DPPH	OH°	O_2_^−^
**1**	50 ± 1.5	85 ± 1.6	62 ± 1.9	80 ± 1.7	92 ± 2	70 ± 1.4	85 ± 1.6	95 ± 2.3	75 ± 1.1
**2**	42 ± 1.2	70 ± 1.5	40 ± 1.3	60 ± 1.5	81 ± 1.9	55 ± 0.9	69 ± 1.2	90 ± 2.5	62 ± 1.4
**3**	35 ± 0.7	67 ± 1.6	55 ± 0.8	58 ± 1.4	75 ± 1.8	62 ± 1.5	63 ± 1.5	79 ± 1.8	68 ± 2.2
**4**	40 ± 0.8	53 ± 1.3	35 ± 0.8	55 ± 1.6	62 ± 2.2	58 ± 2.2	60 ± 2.3	80 ± 1.9	66 ± 2.2
**5**	25 ± 0.7	46 ± 1.4	45 ± 0.8	35 ± 0.8	51 ± 1.3	50 ± 1.3	50 ± 0.8	66 ± 0.8	63 ± 0.9
**AW 00493**	25 ± 0.5	80 ± 1.9	29 ± 0.8	30 ± 0.7	85 ± 1.8	42 ± 0.8	35 ± 0.7	88 ± 1.9	60 ± 1.4
**AW 0597**	15 ± 0.5	88 ± 1.8	35 ± 0.8	19 ± 0.7	90 ± 2.1	47 ± 1.6	30 ± 0.8	95 ± 1.9	70 ± 1.8
**AW 0999**	14 ± 0.4	88 ± 1.8	22 ± 0.7	25 ± 0.4	92 ± 2.6	39 ± 0.8	31 ± 0.5	96 ± 1.9	47 ± 1.3
**BTB 14348**	29 ± 1.6	95 ± 1.8	22 ± 1.6	33 ± 2.2	96 ± 2.1	33 ± 1.3	42 ± 0.8	98 ± 1.9	45 ± 1.6
**HTS 01162**	12 ± 0.7	85 ± 1.9	18 ± 1.6	19 ± 0.7	95 ± 1.9	30 ± 0.5	32 ± 1.3	98 ± 1.8	49 ± 1.4
**HTS 04428**	13 ± 0.5	82 ± 1.9	12 ± 0.5	18 ± 0.8	92 ± 1.4	24 ± 0.8	30 ± 1.3	96 ± 2.1	37 ± 0.8
**HTS 0630**	28 ± 0.8	84 ± 1.8	10 ± 0.3	35 ± 0.9	88 ± 1.8	18 ± 0.7	42 ± 1.3	91 ± 2.5	40 ± 1.5
**JFD 2217**	19 ± 0.7	92 ± 1.9	10 ± 0.5	23 ± 0.7	94 ± 1.9	12 ± 0.3	27 ± 0.8	98 ± 2.1	20 ± 0.8
**NSC 1881**	12 ± 0.4	88 ± 1.9	24 ± 0.6	15 ± 0.4	90 ± 1.8	33 ± 0.8	30 ± 0.8	95 ± 1.8	57 ± 1.3
**NSC 2541**	25 ± 0.8	91 ± 1.8	15 ± 0.5	38 ± 1.4	95 ± 2.5	25 ± 0.7	49 ± 1.4	98 ± 2.1	51 ± 1.5
**NSC 3028**	28 ± 0.8	89 ± 1.5	22 ± 0.4	33 ± 0.6	90 ± 1.9	28 ± 0.8	48 ± 1.4	92 ± 2.1	38 ± 1.4
**NSC 3055**	12 ± 0.3	82 ± 2.1	10 ± 0.2	18 ± 0.5	92 ± 2.1	22 ± 0.7	25 ± 0.8	92 ± 1.8	35 ± 1.2
**NSC 3073**	9 ± 0.2	85 ± 1.8	25 ± 0.8	12 ± 0.3	93 ± 1.9	38 ± 0.8	15 ± 0.3	94 ± 1.9	40 ± 0.8
**NSC 412**	26 ± 0.8	79 ± 1.4	25 ± 0.6	35 ± 0.9	85 ± 1.8	32 ± 1	48 ± 1.4	87 ± 1.9	45 ± 2.2
**NSC 740**	25 ± 0.8	96 ± 1.9	39 ± 1.4	32 ± 1.4	98 ± 2.1	45 ± 1.5	40 ± 1.3	99 ± 1.9	60 ± 1.4
**NSC 996**	15 ± 0.5	80 ± 2.1	15 ± 0.4	28 ± 0.8	82 ± 1.9	30 ± 0.7	40 ± 0.9	85 ± 2.1	55 ± 2.2

∗RSA: Radical Scavenging Activity.

**Table 3:** Description and fit value of pharmacophore mapping of compounds **8a–h.**

Compounds	R	R1	R2	R3	Fit value
**8a**	H	2-OH	4-OH	H	3.2
**8b**	H	2-OH	5-CH3	H	3.0
**8c**	H	2-OH	5-OCH3	H	3.3
**8d**	H	2-Cl	3-NO2	6-Cl	2.6
**8e**	CH3	2-Cl	3-NO2	6-Cl	2.8
**8f**	CH3	2-OH	5-CH3	H	2.7
**8g**	CH3	2-OH	5-OCH3	H	3.1
**8h**	CH3	2-NO2	H	H	2.7

Fit value represents a good match of features of the pharmacophore model with the ligand.

**Table 4:** DPPH radical and Superoxide radical scavenging of synthetic compounds.

Compounds	Concentration *μ*mol·L^ −1^					
	50 *μ*mol·L^ −1^		100 *μ*mol·L^ −1^		150 *μ*mol·L^ −1^	

**% RSA∗**	DPPH	O_2_^−^	DPPH	O_2_^−^	DPPH	O_2_^−^
**8a**	48 ± 1.2	62.5 ± 1.5	65 ± 1.7	70 ± 2.1	75 ± 2	82 ± 2.5
**8b**	35 ± 1.3	38 ± 1.1	46 ± 1.5	45 ± 1.2	59 ± 1.5	58 ± 1.7
**8c**	52 ± 1.2	54.7	68 ± 1.9	65 ± 1.7	83 ± 2.5	76 ± 2.4
**8d**	30 ± 1.5	24.7 ± 1.4	55 ± 1.7	34.5 ± 1.5	67 ± 1.5	45 ± 1.8
**8e**	33 ± 1.1	3.8 ± 0.1	46 ± 1.2	10 ± 0.2	58 ± 1.7	23 ± 0.9
**8f**	25 ± 0.4	21.6 ± 0.6	35 ± 1.1	31 ± 1.1	50 ± 1.2	43 ± 1.2
**8g**	30 ± 0.5	20.8 ± 0.7	45 ± 1.2	33 ± 1.2	60 ± 1.7	45 ± 1.5
**8h**	13 ± 0.3	36.5 ± 1.1	24 ± 0.9	46 ± 1.4	31 ± 1.4	55 ± 1.6

∗RSA: Radical Scavenging Activity.

## References

[1] Behl C., Moosmann B. (2002). Antioxidant neuroprotection in Alzheimer's disease as preventive and therapeutic approach. *Free Radical Biology and Medicine*.

[2] Coulson D. R., Siobhan B., Cathal J., Passmore P., Johnston J. A. (2004). *β*-Secretase activity in human platelets. *Neurobiology of Aging*.

[3] Zhang H. Y., Yang D. P., Tang G. Y. (2006). Multipotent antioxidants: from screening to design. *Drug Discovery Today*.

[4] Day B. J. (2004). Catalytic antioxidants: a radical approach to new therapeutics. *Drug Discovery Today*.

[5] Kettle A. J., Winterbourn C. C. (1997). Myeloperoxidase: a key regulator of neutrophil oxidant product. *Redox Report*.

[6] Desmarchelier C., Ciccia G., Coussio J. (2000). Recent advances in the search for antioxidant activity in South American plants. *Studies in Natural Products Chemistry*.

[7] Scartezzini P., Speroni E. (2000). Review on some plants of Indian traditional medicine with antioxidant activity. *Journal of Ethnopharmacology*.

[8] Kumar A., Kaundal R. K., Iyer S., Sharma S. S. (2007). Effects of resveratrol on nerve functions, oxidative stress and DNA fragmentation in experimental diabetic neuropathy. *Life Sciences*.

[9] Hall N. K., Chapman T. M., Kim H. J., Min D. B. (2010). Antioxidant mechanisms of Trolox and ascorbic acid on the oxidation of riboflavin in milk under light. *Food Chemistry*.

[10] Aruoma O. I., Halliwell B., Hoey B. M., Butler J. (1989). The antioxidant action of N-acetylcysteine: its reaction with hydrogen peroxide, hydroxyl radical, superoxide, and hypochlorous acid. *Free Radical Biology and Medicine*.

[11] Afanas’ev I. B., Dorozhko A. I., Brodskii A. V., Kostyuk V. A., Potapovitch A. I. (1989). Chelating and free radical scavenging mechanisms of inhibitory action of rutin and quercetin in lipid peroxidation. *Biochemical Pharmacology*.

[12] Mitchell J. H., Gardner P. T., McPhail D. B., Morrice P. C., Collins A. R., Duthie G. G. (1998). Antioxidant efficacy of phytoestrogens in chemical and biological model systems. *Archives of Biochemistry and Biophysics*.

[13] Moret V., Laras Y., Pietrancosta N. (2006). 1,1′-Xylyl bis-1,4,8,11-tetraaza cyclotetradecane: a new potential copper chelator agent for neuroprotection in Alzheimer's disease. Its comparative effects with clioquinol on rat brain copper distribution. *Bioorganic and Medicinal Chemistry Letters*.

[14] Benvenisti-Zarom L., Chempand J., Regan R. F. (2005). The oxidative neurotoxicity of clioquinol. *Neuropharmacology*.

[15] Refouvelet B., Guyon C., Jacquot Y. (2004). Synthesis of 4-hydroxycoumarin and 2,4-quinolinediol derivatives and evaluation of their effects on the viability of HepG2 cells and human hepatocytes culture. *European Journal of Medicinal Chemistry*.

[16] Jacquot Y., Cleeren A., Laios I. (2002). Pharmacological profile of 6,12-dihydro-3-methoxy-1-benzopyrano[3,4-b] [1,4]benzothiazin-6-one, a novel human estrogen receptor agonist. *Biological and Pharmaceutical Bulletin*.

[17] Ismaili L., Nadaradjane A., Nicod L. (2008). Synthesis and antioxidant activity evaluation of new hexahydropyrimido[5,4-c]quinoline-2,5-diones and 2-thioxohexahydropyrimido[5,4-c]quinoline-5-ones obtained by Biginelli reaction in two steps. *European Journal of Medicinal Chemistry*.

[18] Dror O., Shulman-Peleg A., Nussinov R., Wolfson H. J. (2004). Predicting molecular interactions in silico—I. A guide to pharmacophore identification and its applications to drug design. *Current Medicinal Chemistry*.

[19] Lyne P. D., Kenny P. W., Cosgrove D. A. (2004). Identification of compounds with nanomolar binding affinity for checkpoint kinase-1 using knowledge-based virtual screening. *Journal of Medicinal Chemistry*.

[20] Hirashima A., Morimoto M., Ohta H., Kuwano E., Taniguchi E., Eto M. (2002). Three-dimensional common-feature hypotheses for octopamine agonist1-arylimidazolidine-2-thiones. *International Journal of Molecular Sciences*.

[21] Amarowicz R., Pegg R. B., Rahimi-Moghaddam P., Barl B., Weil J. A. (2004). Free-radical scavenging capacity and antioxidant activity of selected plant species from the Canadian prairies. *Food Chemistry*.

[22] Siddhuraju P., Becker K. (2006). The antioxidant and free radical scavenging activities of processed cowpea (Vigna unguiculata (L.) Walp.) seed extracts. *Food Chemistry*.

[23] Chung S. K., Osawa T., Kawakishi S. (1997). Hydroxyl radical-scavenging effects of spices and scavengers from brown mustard (Brassica nigra). *Bioscience, Biotechnology and Biochemistry*.

[24] Nishikimi M., Rao N. A., Yagi K. (1972). The occurrence of superoxide anion in the reaction of reduced phenazine methosulfate and molecular oxygen. *Biochemical and Biophysical Research Communications*.

[25] Ismaïli L., Refouvelet B., Robert J. F. (1999). Synthesis of new pyrazolo[4,3-c]quinolin-3-one derivatives and some oxazolo[4,5-c]quinoline-2,4-diones. *Journal of Heterocyclic Chemistry*.

[26] Hatano T., Kagawa H., Yasuhara T., Okuda T. (1988). Two new flavonoids and other constituents in licorice root: their relative astringency and radical scavenging effects. *Chemical and Pharmaceutical Bulletin*.

[27] Brooks B. R., Bruccoleri R. E., Olafson B. D., States D. J., Swaminathan S., Karplus M. (1983). A program for macromolecular energy, minimization, and dynamics calculations. *Journal of Computational Chemistry*.

[28] Smellie A., Teig S. L., Towbin P. (1995). Poling: promoting conformational variation. *Journal of Computational Chemistry*.

[29] Greene J., Kahn S., Savoj H., Sprague P., Teig S. (1994). Chemical function queries for 3D database search. *Journal of Chemical Information and Computer Sciences*.

